# Setting up JBrowse 2 for Visualizing Genome Synteny

**DOI:** 10.1002/cpz1.70236

**Published:** 2025-12-05

**Authors:** Colin Diesh, Garrett Stevens, Scott Cain, Lincoln Stein, Ian Holmes

**Affiliations:** ^1^ Department of Bioengineering University of California Berkeley California; ^2^ Ontario Institute for Cancer Research Toronto Ontario Canada

**Keywords:** comparative genomics, data visualization, genome browser, synteny, whole‐genome alignment

## Abstract

JBrowse 2 is an open‐source genome browser that provides unique features for visualizing syntenic relationships between multiple genomes. This article describes a protocol for setting up synteny views in JBrowse 2, using an assembly‐to‐assembly whole‐genome alignment example. We detail data preparation steps, including the generation and formatting of whole‐genome alignment data into formats compatible with JBrowse 2's synteny visualization capabilities, and show the GUI‐driven process for setting up interactive synteny views and generating publication‐quality figures. This protocol establishes methods for using JBrowse 2 to explore conserved sequences across multiple genomes. © 2025 The Author(s). Current Protocols published by Wiley Periodicals LLC.

**Basic Protocol**: Using JBrowse 2 to show genomic synteny

## INTRODUCTION

Comparative genomics research often involves identifying and analyzing conserved genomic regions across different species. Such analyses are critical for understanding evolutionary relationships, gene function, and genome organization. JBrowse 2 is a genome browser that includes specialized features for displaying conserved genomic relationships between multiple genomes using “synteny views” (Diesh et al., 2023). JBrowse 2 can load data from whole‐genome alignment (for example, minimap2, MUMmer) and gene homolog–based methods (for example, MCScan).

This protocol focuses on setting up JBrowse 2 synteny views using a small example with whole‐genome alignment of bacterial genomes and follows from a previous protocol (see Current Protocols article: Diesh et al., 2024). This protocol contains the necessary steps for preparing input data for setting up JBrowse 2 synteny views. We generate input data for the synteny view by performing a whole‐genome alignment of several *Helicobacter pylori* genomes using minimap2. Subsequently, we demonstrate the GUI‐driven process for visualizing these prepared datasets. The objective of this protocol is to establish a clear procedure for leveraging JBrowse 2 in comparative genomics research, specifically for the identification and analysis of conserved genomic sequences.

## USING JBrowse 2 TO SHOW GENOMIC SYNTENY

This protocol shows how to load data for the JBrowse 2 synteny view. The JBrowse 2 program does not perform the alignment itself, so we show how to use minimap2 to perform the alignment and load the resulting pairwise alignment format (PAF) file into JBrowse 2. Set up JBrowse 2 to run on an Apache 2 HTTP server on an Ubuntu or Debian Linux machine.

### Necessary Resources

#### Hardware

A simple laptop with 8 GB of RAM and 2 GB of disk space is sufficient to complete the steps in this guide. Note that if you are adapting this protocol to larger eukaryotic genomes, it may be more memory and disk space intensive, particularly when using the minimap2 command, but other commands and JBrowse itself require only modest resources and a web browser.

##### Software

Ubuntu or Debian Linux operating system (Ubuntu 22.04 used at the time of publication)
HTTP access to the Linux system (at http://localhost if the web browser is on the same machine as the server or at a publicly accessible URL like http://yourhost.com that points to the server)
JBrowse CLI (@jbrowse/cli), used to download and prepare the JBrowse 2 instance (JBrowse CLI v3.6.4 used at the time of publication)
Node.js, used as the runtime environment for the JBrowse CLI (Node.js v24.1.0 used at the time of publication)
Tabix (Li, 2011), used for compression of and efficient access to files over the network (tabix v1.21 used at the time of publication)
Samtools (Li et al., 2009), used for preparing indexed FASTA files (samtools v1.21 used at the time of publication)
Minimap2 (Li, 2018), used for computing a whole‐genome alignment (minimap2 v2.27‐r1193 used at the time of publication)
NCBI Datasets (O'Leary et al., 2024) (17.3.0 used at the time of publication; installation instructions available at https://www.ncbi.nlm.nih.gov/datasets/docs/v2/command‐line‐tools/download‐and‐install/)

#### Install JBrowse 2 with the Apache web server

1 Install system dependencies and the “JBrowse CLI”. Run the following commands in the shell (the “export” syntax used here to set the environment variable “OUT” for the web directory assumes bash or sh, the most common UNIX shells):
export OUT=/var/www/html/jbrowse2
sudo apt‐get update
sudo apt‐get install nodejs wget apache2 tabix samtools minimap2 unzip
sudo service apache2 start
**## confirm node.js greater than or equal to v18 is installed**
node --version
sudo npm install ‐g @jbrowse/cli**## confirm that the jbrowse CLI is installed**
jbrowse --version
**## “jbrowse create” downloads and unzips the latest version of JBrowse 2**
jbrowse create tmpdir
sudo mv tmpdir $OUT
  The “jbrowse create” command downloads the latest version of jbrowse‐web.zip from https://github.com/GMOD/jbrowse‐components/releases and unzips it to the target path. The above is an accelerated run‐through of the installation. Please see the prior Current Protocols article (Diesh et al., 2024) for more information about basic JBrowse 2 setup.

#### Load genomes and gene annotations into the JBrowse instance

2 As an example, to look at a comparison between several strains of *H. pylori*, use the “NCBI Datasets” tool to automatically download the reference genome in FASTA format and annotations in GFF3 format for *H. pylori* J99 (GCF_000982695.1), *H. pylori* CHC155 (GCF_025998455.1), and *H. pylori* 26695 (GCF_000307795.1). In a scratch directory, run the following commands:
   

**## download and unzip NCBI genomes**
datasets download genome accession GCF_000307795.1 \
--include gff3,genome --filename hpylori_26695.zip
datasets download genome accession GCF_000982695.1 \
--include gff3,genome --filename hpylori_j99.zip
datasets download genome accession GCF_025998455.1 \
--include gff3,genome --filename hpylori_chc155.zip
unzip ‐o hpylori_j99.zip
unzip ‐o hpylori_26695.zip
unzip ‐o hpylori_chc155.zip
cd ncbi_dataset/data
 
**## rename some of the FASTA files**mv GCF_000982695.1/GCF_000982695.1_ASM98269v1_genomic.fna hpylori_j99.fa
mv GCF_000307795.1/GCF_000307795.1_ASM30779v1_genomic.fna hpylori_26695.fa
mv GCF_025998455.1/GCF_025998455.1_ASM2599845v1_genomic.fna hpylori_chc155.fa
 
**## index FASTA files with “samtools faidx”, generates “fai” files**
samtools faidx hpylori_26695.fa
samtools faidx hpylori_j99.fa
samtools faidx hpylori_chc155.fa
 
**## load indexed FASTA files into jbrowse**
jbrowse add‐assembly hpylori_26695.fa --out $OUT --load copy
jbrowse add‐assembly hpylori_j99.fa --out $OUT --load copy
jbrowse add‐assembly hpylori_chc155.fa --out $OUT --load copy
 
**## minimap2 based whole genome alignment**
minimap2 ‐c hpylori_26695.fa hpylori_j99.fa >26695_vs_j99.paf
minimap2 ‐c hpylori_26695.fa hpylori_chc155.fa >26695_vs_chc155.paf
minimap2 ‐c hpylori_chc155.fa hpylori_j99.fa >chc155_vs_j99.paf
jbrowse make‐pif 26695_vs_j99.paf
jbrowse make‐pif 26695_vs_chc155.paf
jbrowse make‐pif chc155_vs_j99.paf
jbrowse add‐track 26695_vs_j99.pif.gz ‐a hpylori_j99,hpylori_26695 \
--out $OUT --load copy --name “26695 vs J99”
jbrowse add‐track 26695_vs_chc155.pif.gz ‐a hpylori_chc155,hpylori_26695 \
--out $OUT --load copy --name “26695 vs CHC155”
jbrowse add‐track chc155_vs_j99.pif.gz ‐a hpylori_j99,hpylori_chc155 \
--out $OUT --load copy --name “CHC155 vs J99”
 
**## create tabix‐indexed gff3 for gene annotations**
jbrowse sort‐gff GCF_000307795.1/genomic.gff | bgzip >hpylori_26695.gff.gz
jbrowse sort‐gff GCF_000982695.1/genomic.gff | bgzip >hpylori_j99.gff.gz
jbrowse sort‐gff GCF_025998455.1/genomic.gff | bgzip >hpylori_chc155.gff.gz
tabix hpylori_j99.gff.gz
tabix hpylori_26695.gff.gz
tabix hpylori_chc155.gff.gz
 
**# load gene annotations**
jbrowse add‐track hpylori_26695.gff.gz --out $OUT \
--load copy ‐a hpylori_26695 --name “NCBI Genes”
jbrowse add‐track hpylori_j99.gff.gz --out $OUT \
--load copy ‐a hpylori_j99 --name “NCBI Genes”
jbrowse add‐track hpylori_chc155.gff.gz --out $OUT \
--load copy ‐a hpylori_chc155 --name “NCBI Genes”
 
**## create text index for gene name searching**
jbrowse text‐index --out $OUT
 
**## set a default location and tracks to load**
echo '{“name”:“Demo”,“views”:[{“type”:“LinearGenomeView”, \
“init”:{“loc”:“NC_018939.1:391,180. .408,961”,“assembly”:“hpylori_26695”, \
“tracks”:[“hpylori_26695.gff”,“26695_vs_j99.pif”]}}]}' >session.json
jbrowse set‐default‐session --session session.json --out $OUT

#### Launch the JBrowse 2 application and search for a gene in the linear genome view

**Figure 1:**
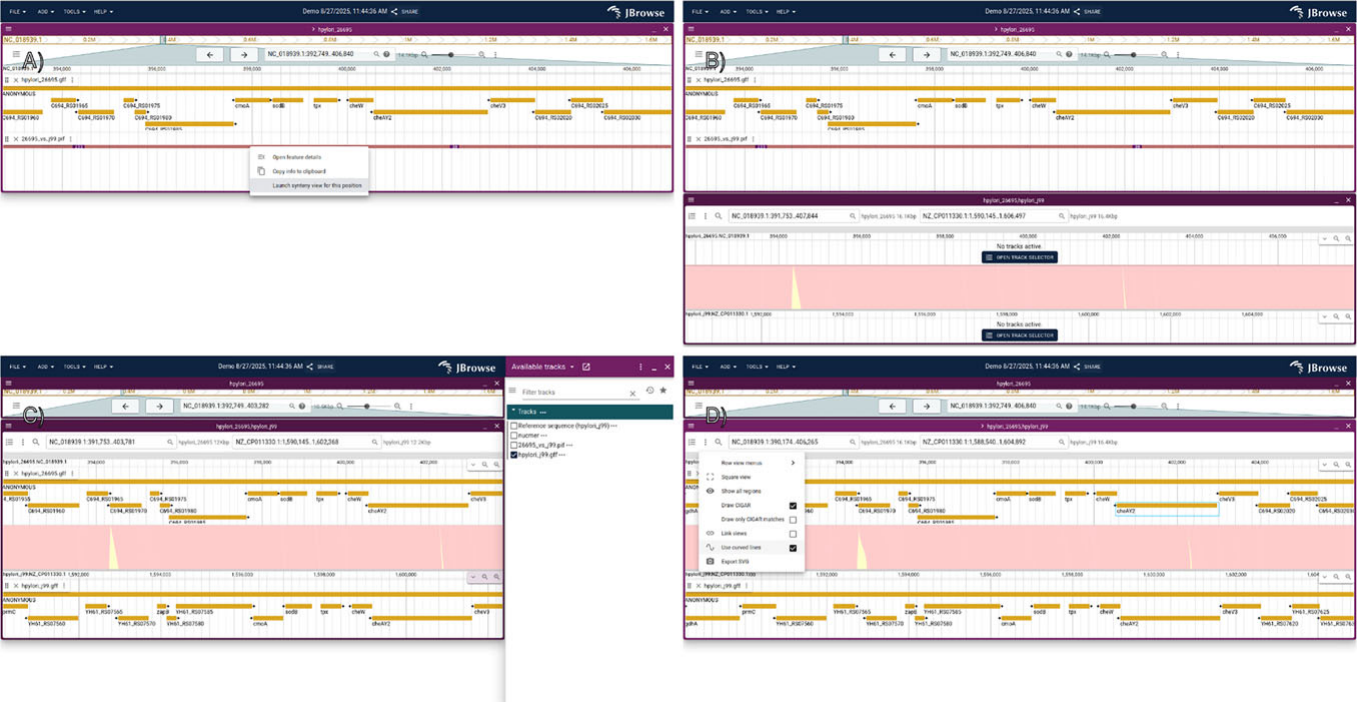
Example procedure for launching the synteny view for a region of interest from a linear genome view. (**A**) Screenshot showing the default session, which shows the *H. pylori* 26695 genome with RefSeq gene annotations and a “synteny track” that represents the minimap2 alignment of *H. pylori* J99 to the *H. pylori* 26695 genome. After right‐clicking an alignment feature, users can select “Launch synteny view for this position”. (**B**) Screenshot showing the result after launching the synteny view. (**C**) Screenshot showing gene tracks opened up on the top and bottom views, which can be accessed from the track selector. (**D**) Screenshot showing additional options, with the “curved” synteny ribbon lines option toggled.

3 Open up the web server, for example, http://yourhost.com/jbrowse2/ (if the server is a public URL) or http://localhost/jbrowse2 (if using the server locally), in a web browser to see a screen that looks something like Figure 1A. Use this classic genome browser view that is shown to visualize synteny by interacting with the “synteny track” (the alignment between the two genomes). See Figure 1 for the detailed procedure.

#### Launch the dotplot view to show a whole‐genome overview of the alignment between two strains

**Figure 2:**
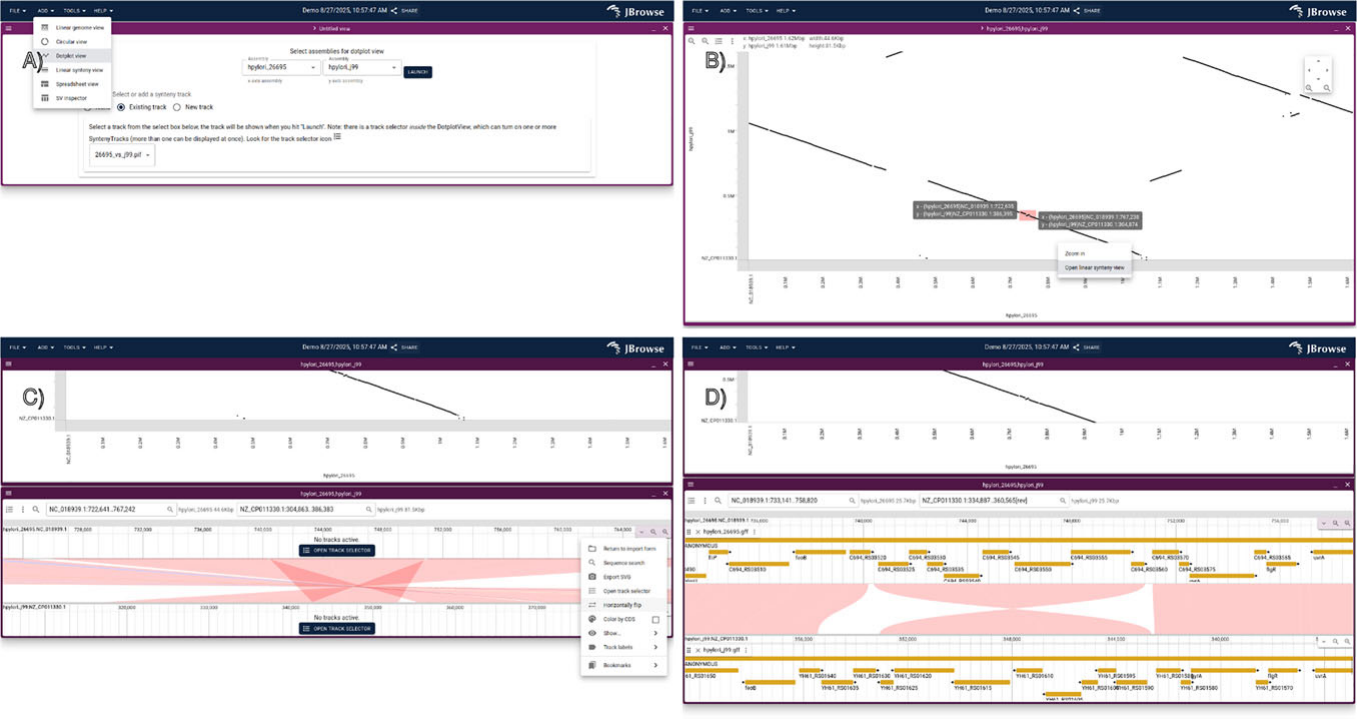
Example procedure for launching the synteny view from the dotplot view to compare two genomes. (**A**) Screenshot of the dotplot view startup form. (**B**) Screenshot of the dotplot view after launch, with additional clicking and dragging a region of interest. (**C**) The result after launching the linear synteny view from the clicked region from (B). (**D**) The result after applying the “Horizontally flip” operation and opening gene tracks.

4 View a whole‐genome overview of the alignment using the dotplot view, which is shown in Figure 2.
  For large eukaryotic alignments, whole‐genome dotplot views can be slow or freeze the browser. See the Troubleshooting section in the Commentary for more information and workarounds.

#### Launch the linear synteny view to show a multi‐way synteny view between three strains

**Figure 3:**
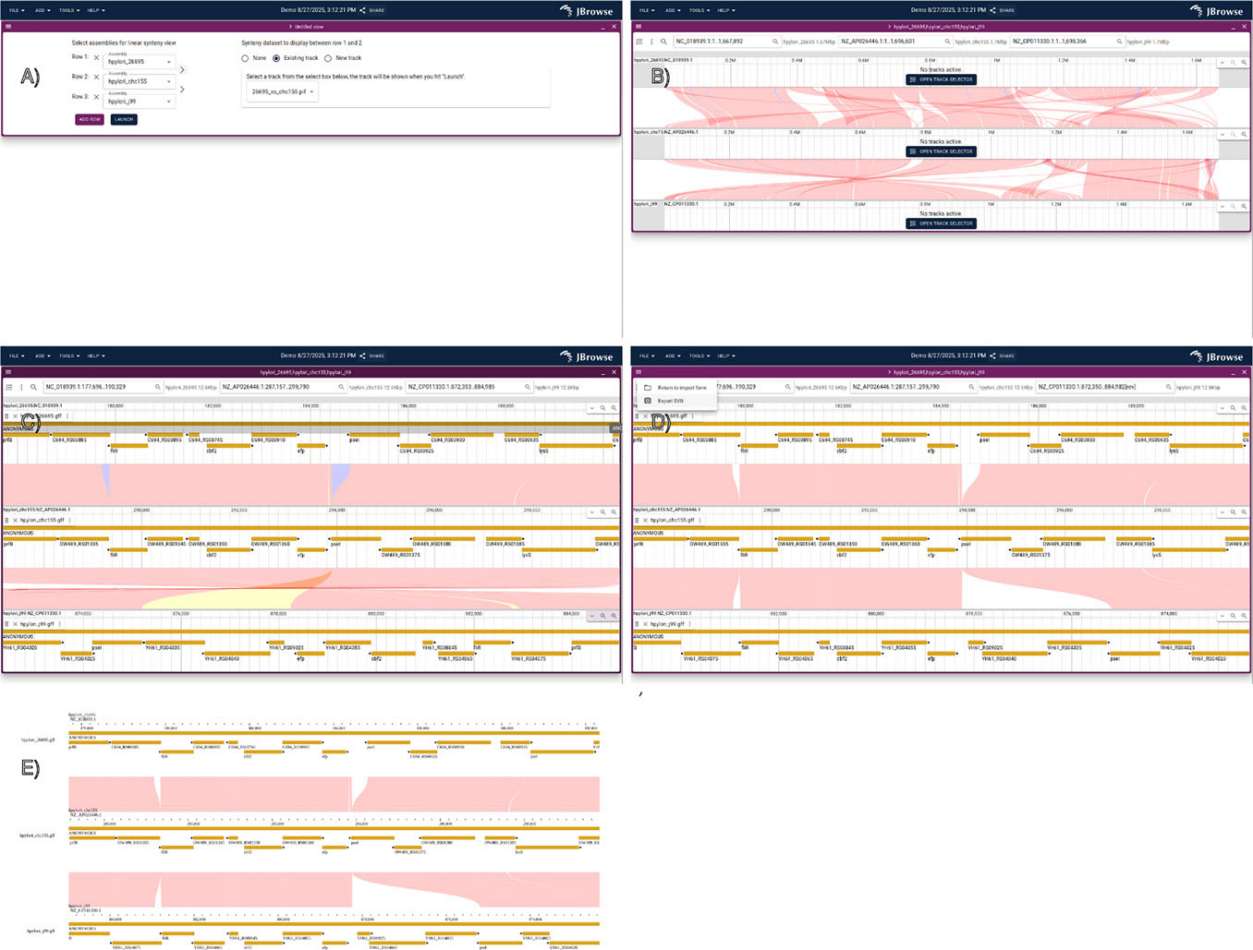
Example procedure for using the linear synteny view to compare three genomes. (**A**) Screenshot showing how to set up the linear synteny view. The “Add row” button is clicked to make three rows visible, and then the synteny dataset to be displayed between each row is chosen. (**B**) Screenshot of the linear synteny view after initial launch, which is a whole‐genome overview of the three genomes. (**C**) Screenshot of the application after searching for the “efp” gene for each view level and then zooming out a little bit to show a larger genomic region around the gene. (**D**) Screenshot showing the result after applying the “Horizontally flip” function to the bottom row (J99) and the “Draw only CIGAR matches” function. It also shows the “Export SVG” menu option open. (**E**) The result of using the “Export SVG” option, which can help generate publication‐quality figures.

5 Launch a linear synteny view to see a whole‐genome overview of synteny. See Figure 3 for detailed instructions on how to launch a multi‐way synteny view of three *H. pylori* strains.
  Similar to the dotplot view in step 4, for large eukaryotic alignments, the whole‐genome synteny overview can be slow or freeze the browser. See the Troubleshooting section in the Commentary for more information and workarounds.
https://gist.github.com/cmdcolin/c8a743f2cb8f1907f14985e155d421af

## COMMENTARY

### Background Information

This protocol outlines a methodology for deploying JBrowse 2 on a web server and incorporates whole‐genome alignment data to visualize genome synteny. These workflows can also be incorporated in the JBrowse 2 desktop application, which runs on Mac, Windows, and Linux, but generally involves using a point‐and‐click GUI to set up. Users are directed to https://jbrowse.org for supplementary documentation, tutorials, and examples.

### Critical Parameters

#### Disk space

Users should have enough disk space to complete operations. Typically, this is approximately 2× the size of your data files because you create a copy with –load copy, but note that –load move and –load symlink do not require making a copy of the data files.

#### Memory

If you are creating your own whole‐genome alignments, particularly on large eukaryotic genomes, it can be very memory intensive, so please review the documentation of the related tools for guidance. We only aligned small bacterial genomes in the above workflow, which requires <2 Gb of memory to run.

### Troubleshooting

See Table 1 for sources of potential errors and their solutions.

**Table 1:** Troubleshooting Guide for Visualizing Genome Synteny with JBrowse 2

Problem	Possible cause	Solution
Your web browser stalls or freezes when viewing synteny datasets	The file for the alignment is too large to be loaded into memory	There are several solutions for this: (a) Filter out low‐quality or small alignment fragments. (b) Remove the CIGAR strings from the minimap2 output by running minimap2 without the “‐c” argument. The CIGAR strings increase the size of the file but provide per‐base information. (c) Use a synteny finding method that focuses on protein‐coding genes rather than whole‐genome sequences, such as MCScan, which produces much less data. (d) Ensure you are using the PIF file format. The “jbrowse make‐pif” command generates a pif.gz file from a PAF file, which can then be loaded with “jbrowse add‐track”.
The linear synteny view or dotplot view is “blank” or “empty”	The assembly names are not specified correctly; for example, they are flipped	If you run “minimap2 ref.fa query.fa >out.paf”, then run “jbrowse add‐track ‐a query, ref” (the order of the assemblies in ‐a matters).
There is no area for showing synteny between views in the linear synteny view	The user did not choose a dataset for the synteny ribbons to show	At the import form (e.g., in Figure 3A), click the “>” button between the two rows and choose a synteny dataset, or open the “Synteny track selector”.
‍You get error messages regarding node.js or have the wrong version of node.js installed	The apt repository installed the wrong version of node.js for you	You can run “sudo apt‐get purge ‐y nodejs npm” to remove your node.js install. Then, please visit https://nodejs.org/en/download for non‐apt instructions for installing node.js, or https://nodesource.com/products/distributions for apt instructions.

### Understanding Results

After completing the protocol steps, your JBrowse 2 instance will be live and accessible via a URL like http://yourhost.com/jbrowse2/, assuming public access to that address. This location stems from creating the jbrowse2 folder within /var/www/html/ (the default web root directory for Apache on Ubuntu). Feel free to relocate or rename this folder to suit your needs.

Note that accurately creating whole‐genome alignments is a complex task in and of itself that may vary depending on your species of interest. For small bacterial genomes, using minimap2 with default parameters may work well, but for large, complex eukaryotic genomes, you may need specialized parameters or pipelines. We recommend investigating the best practices and options regarding whole‐genome alignment for your species of interest.

JBrowse 2 can load data from MUMmer (which outputs .delta files), UCSC liftOver (which outputs .chain files), MashMap (which creates PAF files), or MCScan (which creates .anchors for homologous genes and .anchors.simple for syntenic blocks). For more documentation on setting up synteny datasets, see our documentation page (JBrowse, n.d.). We recommend checking the JBrowse 2 documentation to see the most up‐to‐date information.

If you see any errors during your setup, please take note of the error message and report it to the JBrowse 2 GitHub page, at https://github.com/GMOD/jbrowse‐components, to get support from the JBrowse 2 team. A script containing all the data preparation steps and the expected output of running the commands on the command line can be found here: https://gist.github.com/cmdcolin/c8a743f2cb8f1907f14985e155d421af.

### Time Considerations

Users can comfortably complete the Basic Protocol within a couple of hours. The whole‐genome alignment step takes <1 min for the *H. pylori* genomes used in this article. Larger eukaryotic genomes can take hours to days to align properly with minimap2, so be aware of this. Alternative tools such as MCScan, which focuses on aligning the protein‐coding genes instead of the whole‐genome sequence, can also be used to reduce runtime.

### Author Contributions

**Colin Diesh**: Conceptualization; software; visualization; writing—original draft; writing—review and editing. **Garett Stevens**: Software; validation; visualization; writing—original draft; writing—review and editing. **Scott Cain**: Software; validation; writing—review and editing. **Lincoln Stein**: Project administration; supervision; writing—review and editing. **Ian Holmes**: Conceptualization; funding acquisition; project administration; supervision; writing—original draft; writing—review and editing.

### Conflict of Interest

The authors declare no conflict of interest.

## Data Availability

The data that support the findings of this study are available in NCBI RefSeq at https://www.ncbi.nlm.nih.gov/refseq/. These data were derived from the following resources available in the public domain: *H. pylori* J99 (GCF_000982695.1), *H. pylori* CHC155 (GCF_025998455.1), and *H. pylori* 26695 (GCF_000307795.1).
